# Supplementary material for: Deployed Veterans exhibit distinct respiratory patterns and greater dyspnea during maximal cardiopulmonary exercise: A case-control study
Source: PLoS One. 2023 May 24;18(5):e0286015. doi: 10.1371/journal.pone.0286015 (PMC10208524; doi:10.1371/journal.pone.0286015)
Supplement: Attachment — Submitted filename: Response to Reviewers.docx [file pone.0286015.s001.docx]

**Response to Reviewers**

**Thank you to the editor and reviewers for taking the time to review and provide feedback on our work. We believe that the revised manuscript has been strengthened through this process and hope that our responses to your comments are to your satisfaction.**

**Editor:**

1. Please ensure that your manuscript meets PLOS ONE's style requirements, including those for file naming. The PLOS ONE style templates can be found at

<https://journals.plos.org/plosone/s/file?id=wjVg/PLOSOne_formatting_sample_main_body.pdf>

and <https://journals.plos.org/plosone/s/file?id=ba62/PLOSOne_formatting_sample_title_authors_affiliations.pdf>.

**Thank you. We have closely reviewed these guidelines and formatted our documents accordingly.**

2. We note that the grant information you provided in the ‘Funding Information’ and ‘Financial Disclosure’ sections do not match. When you resubmit, please ensure that you provide the correct grant numbers for the awards you received for your study in the ‘Funding Information’ section.

**Done. Thank you.**

3. Thank you for stating the following in the Acknowledgments Section of your manuscript:

“The authors would like to thank the volunteers who participated in this study as well as data collection and analysis support from Bishoy Samy and Nancy Eager. This work was supported by Pilot Project Award # I21RX001079 from the United States (U.S.) Department of Veterans Affairs Rehabilitation Research and Development Service and supported in part by Merit Review Award # I01CX001515 and Career Development Award # IK2CX001679 from the U.S. Department of Veterans Affairs Clinical Sciences Research and Development Service. This study was registered on clinicaltrials.gov (NCT01754922). The contents do not represent the views of the U.S. Department of Veterans Affairs or the United States Government.”

We note that you have provided funding information that is not currently declared in your Funding Statement. However, funding information should not appear in the Acknowledgments section or other areas of your manuscript. We will only publish funding information present in the Funding Statement section of the online submission form.

Please remove any funding-related text from the manuscript and let us know how you would like to update your Funding Statement. Currently, your Funding Statement reads as follows:

“Include this sentence at the end of your statement: The funders had no role in study design, data collection and analysis, decision to publish, or preparation of the manuscript.”

Please include your amended statements within your cover letter; we will change the online submission form on your behalf.

**We apologize for this oversight. Funding information is now removed from the Acknowledgements section of the manuscript. Thank you for changing the online submission form on our behalf. The cover letter contains the requested language to be used in the Funding Statement.**

4. In your Data Availability statement, you have not specified where the minimal data set underlying the results described in your manuscript can be found. PLOS defines a study's minimal data set as the underlying data used to reach the conclusions drawn in the manuscript and any additional data required to replicate the reported study findings in their entirety. All PLOS journals require that the minimal data set be made fully available. For more information about our data policy, please see <http://journals.plos.org/plosone/s/data-availability>.

Upon re-submitting your revised manuscript, please upload your study’s minimal underlying data set as either Supporting Information files or to a stable, public repository and include the relevant URLs, DOIs, or accession numbers within your revised cover letter. For a list of acceptable repositories, please see http://journals.plos.org/plosone/s/data-availability#loc-recommended-repositories. Any potentially identifying patient information must be fully anonymized.

Important: If there are ethical or legal restrictions to sharing your data publicly, please explain these restrictions in detail. Please see our guidelines for more information on what we consider unacceptable restrictions to publicly sharing data: http://journals.plos.org/plosone/s/data-availability#loc-unacceptable-data-access-restrictions. Note that it is not acceptable for the authors to be the sole named individuals responsible for ensuring data access.

We will update your Data Availability statement to reflect the information you provide in your cover letter.

**Thank you for raising this concern. We apologize for this omission. The data availability statement is now included in the cover letter and explains the ethical/legal restrictions for why a minimal underlying dataset was not uploaded (similar to our prior publication in PLOS ONE** <https://doi.org/10.1371/journal.pone.0224833>**).**

**Reviewer #1:**

1. This study shoes that compared to non-deployed controls, Veterans deployed to Southwest Asia (SWA) had reduced respiratory frequency and greater dyspnea during maximal exercise. Associations of these parameters were found only in deployed Veterans. These findings, despite some limitations recognized by the authors, provide support for an association between deployment to SWA and respiratory health and highlight the utility of respiratory frequency in the clinical evaluation of deployment-related dyspnea in Veterans. The study was well conducted and is well written. The only changes I suggest consist in the utility of providing more information for the average readers and on data from Veterans in previous conflicts in Asia, for example in Afghanistan, which involved many countries such as the USA, various European countries, Canada and Australia

**Thank you. In response to your comment about referring to earlier conflicts in this region and a related comment from reviewer 2, we have added the following to the manuscript (Lines 98-100).**

**“We have previously found that the components of exercise V̇E afforded insight into evaluating exercise intolerance among deployed veterans of earlier miliary conflicts (13).”**

**Reviewer #2:**

1. Title needs to be modified. “Name the trial design” should appear in the title.

**Thank you. The title now reads, “Deployed Veterans exhibit distinct respiratory patterns and greater dyspnea during maximal cardiopulmonary exercise: A case-control study”**

**The study design has also been added to the Methods section.**

2. All figures aren’t clear, please repeat the figures to be more clear and readable

**Thank you for this comment. We agree that the figures are blurry in the PDF that is generated in the PLOS ONE manuscript portal. However, we have ensured that they meet the journal’s requirements for resolution. Please note that clicking on the hyperlink in the top right corner of each figure page will allow you to download a higher quality image than shown in the PDF (e.g.** [**https://www.editorialmanager.com/pone/download.aspx?id=31706934&guid=53852b6a-7b61-42ee-aad9-c4c4799c0cd4&scheme=1**](https://www.editorialmanager.com/pone/download.aspx?id=31706934&guid=53852b6a-7b61-42ee-aad9-c4c4799c0cd4&scheme=1)**). We will defer to the editor for further guidance on this matter if needed.**

3. Line 99 (Hence, the purpose of this study) is not clear –please clarify what’s the novelty of your study –why you measure CPET for SWA deployed Veterans relative to non-deployed control (what is known and what’s unknown )

**Thank you. We have now revised this last paragraph to succinctly clarify the empirical rationale for using CPET:**

**“We have previously found that the components of exercise V̇E afforded insight into evaluating exercise intolerance among deployed veterans of earlier miliary conflicts [13].”**

4. As you point out in line 107 your study was an observational pilot study so please add STROBE Checklist

**Thank you for this important point. While a majority of the applicable STROBE checklist items were already incorporated in the original submission, the revised version contains additional items that were not included (e.g., study design in the title; describing the setting, locations, and relevant dates).**

5. Line 160 (Blood pressure was manually) how it done manually during Bruce protocol (maybe have false reading) did you consider these error during the study period

**Our laboratory – trained clinical exercise physiologists - routinely perform manual auscultation of blood pressure throughout the exercise protocol per our safety protocols. In our experience, manual auscultation of blood pressure is less prone to error than automated machines during exercise. Blood pressure was not an outcome of interest for this study or included in analyses. Therefore, we are not concerned that error associated with blood pressure measurement will impact our results.**

6. In Table 1. Participant demographics, self-reported health, and physical presented, you listed the smoking status of the participant only. Is this the only factor that will affect the physical health of the participants? You did not put in the inclusion or exclusion criteria, so we need more focus in that point.

**We did not exclude for smoking but recognize this may be a factor that influences cardiorespiratory performance in this population. There are likely many factors that influence physical health and considering all potential influences on health was beyond the scope of this study. Because this study is focused on lung function, we felt that smoking status was important to consider.**

7. The discussion section needs to be described scientifically. Kindly frame it along the following lines:
   a. 1-Main findings of the present study
   b. 2-Comparison with other studies
   c. 3- Implication and explanation of findings

**We agree that the above elements are important to include in a discussion section. At the same time, we feel that each element is already provided and would like to bring your attention to the specific sections in which they are located.**

***Main findings*:**

**“The primary aim of this study was to evaluate the potential utility of CPET to provide physiological insight into respiratory symptoms reported by Veterans deployed to SWA (i.e., dyspnea). Specifically, we tested whether SWA deployment is associated with deficiencies in cardiorespiratory health by comparing physiological and perceptual responses to maximal exercise between deployed Veterans and non-deployed controls. After restricting our analytic dataset to 36 participants (25 deployed, 11 non deployed) meeting criteria for a valid peak effort (Fig 1), we observed minimal between-group differences for a majority of CPET variables (V̇E, V̇CO_2_, V̇O_2_, V_T_, RER, and HR). However, we observed moderate-large group (η^2^_partial_ =0.26) and group-by-time interaction (η^2^_partial_ =0.096) effects for *f*_R_. As shown in Fig 2, these findings are characterized by slower *f*_R_ and a greater change over time in deployed Veterans relative to non-deployed controls.”**

***Comparison with other studies***:

**“Our findings can be interpreted in the context of prior research involving both healthy adults and deployment-related illnesses such as Gulf War Illness (GWI). Consistent with earlier work which found that V_T_ is determined more by metabolic factors than *f*_R_ [31–34], a study of healthy adults found that *f*_R_, but not V_T_, responds rapidly to changes in workload during high-intensity cycling and recovery, is independent from metabolic factors (V̇CO_2_, V̇O_2_), and is strongly associated with RPE [35]. Further, our prior study of Veterans with GWI found altered breathing patterns in Veterans with GWI, as indicated by higher V_T_ and lower *f*_R_ compared to non-symptomatic controls [13]. Additionally, group differences in *f*_R_ were larger than V_T_, suggesting that V̇E was primarily driven by *f*_R_ in symptomatic Veterans.”**

**“Following our observation of differential *f*_R_ patterns between deployed and non-deployed participants, we conducted exploratory correlational analyses which revealed that dyspnea ratings were significantly associated with *f*_R_ at 80% (r=0.58) and 100% (r= 0.41) of V̇O_2peak_, but only in deployed Veterans (Figs 3-4). The design of the present study does not lend itself to determining whether there is a mechanistic link between *f*_R_ and dyspnea, but we are not the only group to observe an association between these two variables. For instance, in healthy young adults who completed a staged cycle ergometry protocol (50W/4min), Tsukada reported that the threshold at which *f*_R_ becomes tachypneic is preceded by and associated with the point at which exertional dyspnea begins to rapidly increase [36].”**

**“Multiple other studies have conducted CPET in deployed SWA military personnel and Veterans [1,3,7,11,12]; however, analyses have focused primarily on traditional parameters (e.g., peak exercise) and do not consider dynamic exercise ventilation patterns. For instance, two separate studies by Morris and colleagues report numerous CPET values but limited to two timepoints: V̇O_2peak_ and VAT [3,7]. Interestingly, authors observed increased peak *f*_R_ among those with dyspnea relative to controls (Mean (SD): 50.2 (12.4) vs 44.5 (6.7)) but it is unclear whether *f*_R_ differences persisted at submaximal intensities. Moreover, other studies that have included CPET in their analyses [1] only presented V̇O_2peak_ and percent abnormal for V̇O_2peak,_ V̇E/MVV, V̇E/V̇CO_2_, and VAT. Similar to the present study, these previously performed studies rarely identify between-group differences when restricting CPET analyses to traditional indices. It should be noted that unlike the present sample comprised of non-treatment seeking Veterans, previously published studies in military personnel and Veterans underwent CPET as part of a clinical evaluation for respiratory complaints [1,3–5,12,37]. Although the present study sample was not referred for clinical evaluation, the deployed Veteran group endorsed considerable respiratory symptom burden (Table 2).”**

***Implications and explanations of findings:***

**“Integrating these prior studies with the present study, our observation of slower *f*_R_ in deployed Veterans may represent a centrally-mediated, learned strategy to mitigate dyspnea symptoms during exercise (i.e., breathing slower may help decrease feelings of breathlessness). However, it is important to note that we did not collect data on breathing patterns prior to development of dyspnea symptoms, so longitudinal CPET studies assessing pre- and post-deployment health are clearly needed to establish temporal sequence.”**

**“The investigators speculated that unpleasantness accompanied by dyspnea reaches a level which induces emotional respiratory reactions to stimulate a tachypneic breathing pattern. However, our findings are somewhat counterfactual to that interpretation. That is, despite reporting higher dyspnea ratings throughout the CPET, deployed Veterans had slower *f*_R_ values than non-deployed Veterans at every timepoint (Table 6, Fig 2), as also observed in our prior work involving Veterans with Gulf War Illness [13]. Given that Tsukada focused on healthy adults whereas we focused on Veterans with deployment related exposures, perhaps people who experience significant respiratory symptoms on a day-to-day basis are less susceptible to dyspnea-driven increases in *f*_R_ because they are more familiar with the experience of dyspnea than otherwise healthy people (Table 2). This hypothesis could be tested by studying the effect of experimentally manipulated dyspnea on *f*_R_ in a direct comparison of healthy and symptomatic Veterans.”**

**“In our sample, Veterans deployed to SWA exhibit reduced *f*_R_ and greater dyspnea during maximal exercise relative to non-deployed controls. Further, *f*_R_ is positively associated with dyspnea ratings at 80% and 100% of V̇O_2peak_ in deployed Veterans but not in non-deployed controls. These findings provide support for a potential association between deployment to SWA and cardiorespiratory health. In addition, our findings highlight the utility of incorporating CPET for the evaluation of exertional dyspnea beyond that of traditional peak indices to investigate the dynamic behavior of exercise ventilation.”**
