# Supplementary material for: Deployed Veterans exhibit distinct respiratory patterns and greater dyspnea during maximal cardiopulmonary exercise: A case-control study
Source: PLoS One. 2023 May 24;18(5):e0286015. doi: 10.1371/journal.pone.0286015 (PMC10208524; doi:10.1371/journal.pone.0286015)
Supplement: Attachment — Submitted filename: Response to Reviewers.docx [file pone.0286015.s002.docx]

**Response to Reviewers**

**Thank you for the additional feedback. We are delighted to hear that the manuscript only required minor revisions at this stage. Please see our responses to your comments below.**

1. Some references are very old (22, 23,24, 29). Please update the references.

**If possible, we would prefer to keep these specific references as they are the original source for the corresponding method that we are describing in that section.**

2. Change the subheading "introduction" to "Background" in accordance with the guidelines. In the abstract, remove the hypothesis.

**Done.**

3. Mention the level of significance in the results.

**Done. We have also added P-values to the tables where appropriate.**
